# Almonertinib-induced interstitial lung disease in an NSCLC patient co-harboring EGFR Ex19del mutation and MET *de novo* amplification: a case report and literature review

**DOI:** 10.3389/fonc.2025.1481244

**Published:** 2025-02-17

**Authors:** Wenjing Yang, Lin Shi, Hao Wang, Ying Li, Xingyu Ji, Hongjin Li, Guowang Yang, Weiru Xu

**Affiliations:** ^1^ Beijing Hospital of Traditional Chinese Medicine, Capital Medical University, Beijing, China; ^2^ School of Clinical Medicine, Beijing University of Chinese Medicine, Beijing, China

**Keywords:** non-small cell lung cancer (NSCLC), Ex19del mutation and MET *de novo* amplification, almonertinib, interstitial lung disease (ILD), case report

## Abstract

Lung cancer patients co-harboring EGFR Ex19del mutation and MET *de novo* amplification is extremely uncommon. Thus, the optimal therapeutic strategies, treatment-related complications, and prognosis for such patients remain unclear. Herein, we describe a case of patient co-harboring EGFR Ex19del mutation and MET *de novo* amplification who presented targeted (almonertinib)-induced interstitial lung disease (ILD). We propose that patients with EGFR Ex19del mutation and MET *de novo* amplification may benefit more from dual-targeted therapy than pemetrexed and carboplatin chemotherapy along with bevacizumab. However, dual-targeted therapy may increase the risk of ILD, so it is important to be alert to targeted-induced ILD, and unexplained fever may be an early warning signal for targeted-induced ILD, especially almonertinib-induced ILD. Timely intervention is needed to avoid greater harm when ILD occurs and, when ILD is effectively controlled, seize the opportunity to rechallenge the dual-targeted therapy may contribute to a better prognosis. In addition, the patients with targeted-induced ILD in the past need more rigorous monitoring and follow-up in the process of rechallenging the targeted drug therapy.

## 1 Introduction

The incidence and mortality of lung cancer have increased globally in recent years. Moreover, non-small cell lung cancer (NSCLC) is the most common histological type, which accounts for 80%–85% of all lung carcinomas (1, 2). In the past decade, targeted therapies have revolutionized the treatment and improved the outcome for oncogene-driven NSCLC (3). Moreover, the epidermal growth factor receptor (EGFR) is one of the most common driver genes in NSCLC, which occur in 10% to 15% of the western population and 40% to 60% of the Asian population. What is more, in women and non-smoking Chinese people, the EGFR-sensitive mutation rate is even higher (4–6).

MET amplification as a *de novo* driver alteration occurring in NSCLC patients is not high (1%–5% of untreated NSCLC) (7, 8), which is always strongly associated with smoking. However, MET amplification has emerged as a significant mechanism of acquired resistance in various targeted therapies (5%–22%), such as EGFR mutation, KRAS G12C mutation, ALK fusion, ROS1 fusion and RET fusion, and particularly in EGFR-mutant NSCLC (7–17).

To our knowledge, the report about lung cancer patient co-harboring EGFR Ex19del mutation and MET *de novo* amplifications is extremely uncommon (18), and a unified standard treatment plan has not been formed yet. However, the certain thing is that the first-line dual-targeted regimens are not routinely recommended for this group of patients. For this reason, the experience related to dual-targeted therapy is not rich, and the experience about diagnosis and management of the toxic side effects, such as ILD, which is induced by targeted therapy for this group of patients, is relatively lacking. In addition, ILD induced by the combination of almonertinib (targeted to EGFR Ex19del mutation) and glumetinib (targeted to MET *de novo* amplifications) has not been reported. Thus, accumulating relevant experience in this field is necessary.

Herein, we report a case of lung adenocarcinoma co-harboring Ex19del mutation and MET *de novo* amplification. The patient got successful remission of ILD, which was induced by almonertinib. Up to now, the rechallenge of dual-targeted therapy (furmonertinib and glumetinib) is more than 2 months without recurrence of ILD.

## 2 Case presentation

**Figure 1:**
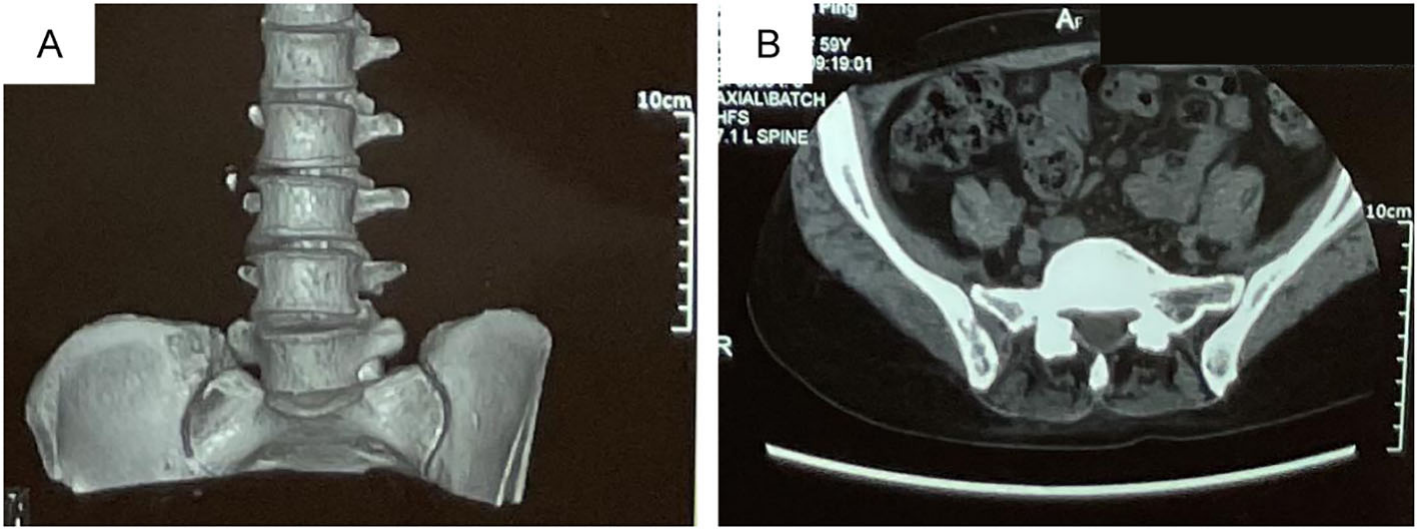
The right sacroiliac joint bone destruction with soft tissue mass (malignant?) was found by lumbar computed tomography (CT) scan on May, 11, 2023. **(A)** Three-dimensional reconstruction of the right sacroiliac joint. **(B)** Coronal CT scan of the right sacroiliac joint. The patient, a 60-year-old female with no history of smoking, had no prior medical conditions, with a chief complaint of the pain in the right lumbosacral region and was found the right sacroiliac joint bone destruction with soft tissue mass (malignant?) by lumbar computed tomography (CT) scan on May, 11, 2023 **(A, B)**.

**Figure 2:**
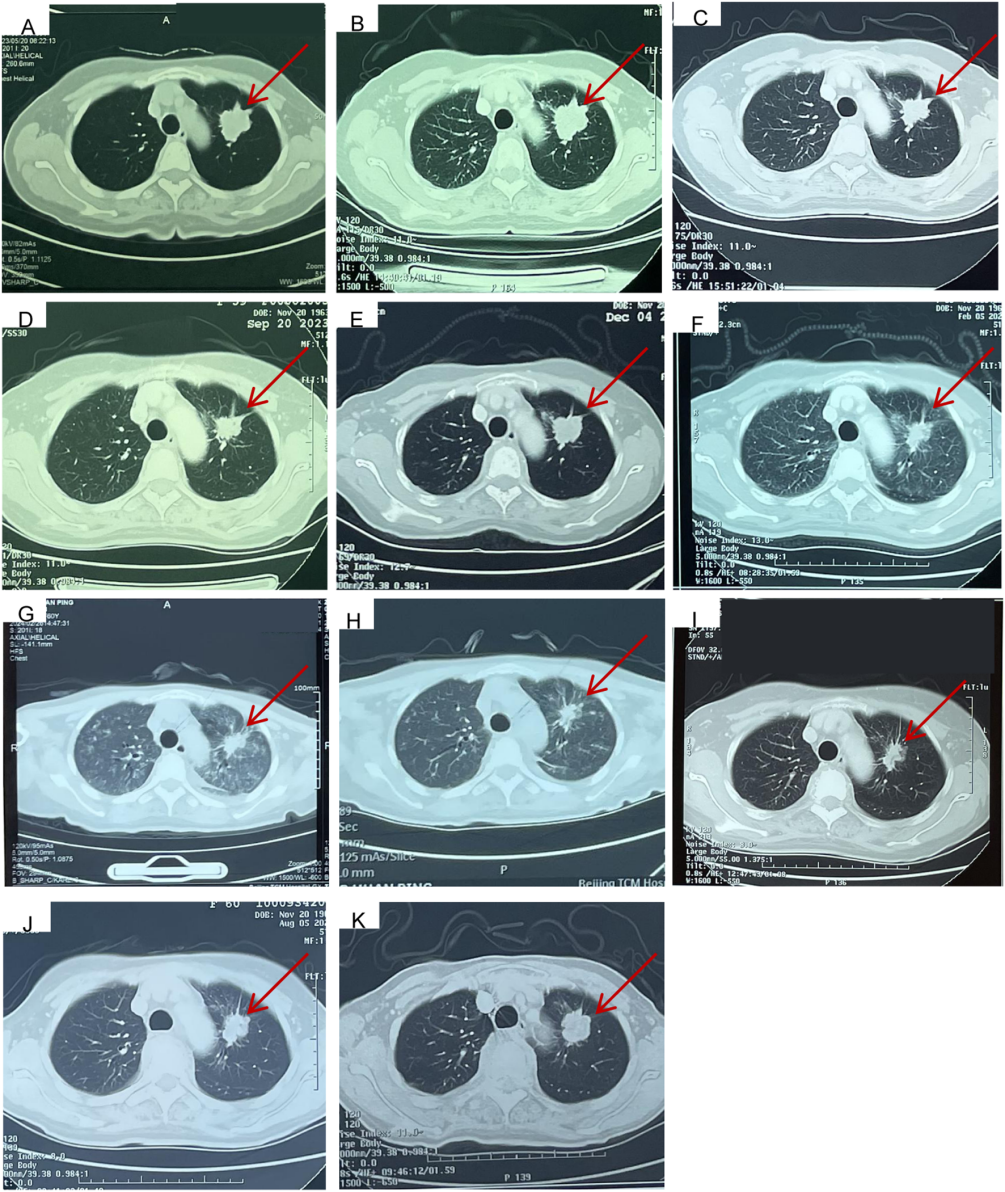
Computed tomography findings. **(A)** Primary lesion (26*37mm) in upper left lung at the time of first visit to Cancer Hospital, Chinese Academy of Medical Sciences on May, 20, 2023. **(B)** Baseline image (Primary lesion 29*36mm) before taken front-line pemetrexed and carboplatin chemotherapy along with bevacizumab targeted therapy at Jun, 15, 2023. **(C)** Response evaluation (Primary lesion 29*22mm, SD) after taken 2 cycles of pemetrexed and carboplatin chemotherapy along with bevacizumab targeted therapy at Jul, 24, 2023. **(D)** Response evaluation (Primary lesion 29*22mm, SD) after taken 4 cycles of pemetrexed and carboplatin chemotherapy along with bevacizumab targeted therapy at Sept, 20, 2023. **(E)** Twenty-one days after taken almonertinib combined with glumetinib targeted therapy (Primary lesion 30*28mm, SD) at Dec, 5, 2023. **(F)** eighty-four days after taken almonertinib combined with glumetinib targeted therapy (Primary lesion 26*18mm, PR) at Feb, 5, 2024. **(G)** The emergency chest CT indicated diffuse interstitial lung disease (ILD) at Feb, 26, 2024, (Primary lesion 28*20mm, SD). **(H)** The ILD has been well controlled at Mar, 4, 2024, (Primary lesion 28*20mm, SD). **(I)** twenty-two days after the rechallenge of the dual-targeted therapy at May, 7, 2024, (Primary lesion 28*19mm, SD). **(J)** Response evaluation (Primary lesion 28*19mm, SD) at Aug, 5, 2024. **(K)** Response evaluation (Primary lesion 28*25mm, SD, but showing a slow progress trend) at Nov, 11, 2024.

The patient, a 60-year-old woman with no history of smoking, had no prior medical conditions, with a chief complaint of the pain in the right lumbosacral region, and in the right sacroiliac joint, bone destruction with soft tissue mass (malignant)? was found by lumbar computed tomography (CT) scan on 11 May 2023 (**Figures 1A, B**). Then, further workup was completed showing a 37 mm × 36 mm lesion (lung cancer)? in the upper left lung by chest CT on 20 May 2023 (**Figure 2A**). 12 days after the first visit, a sacroiliac bone (right side) biopsy was performed. The pathological results, in conjunction with immunohistochemistry findings, indicated TTF-1(+), CK7(+), Naspsin A(+), ALK(Ventana)(−), CK20(−), and PDL1(SP263) (TPS:0). Unfortunately, the first diagnosis was left lung adenocarcinoma with bone metastasis. PET-computed tomography (PET-CT) indicated multiple pulmonary, liver, bone, and lymph node (lung hilum, mediastinum) metastases, staging IV (cT2aN2M1). Meanwhile, molecular screening of sacroiliac bone-biopsy-tumor-tissue by a large gene new-generation sequencing (NGS) panel analysis identified EGFR 19 exon p.L747-P753 delinsS (33.9%), EGFR 19 exon p.L747S mutation (0.90%), TP53 exon8 p.V272L mutation (34.17%), MET (CN=9), CCNE1 (CN=11), RICTOR (CN=9), ATK3 (CN=9), CDK6 (CN=9), HGF (CN=9) amplification, microsatellite stable, and a level of 1.67Mut/Mb in tumor mutation burden.

The patient received six cycles of front-line pemetrexed and carboplatin chemotherapy along with bevacizumab-targeted therapy and bisphosphonate bone protection treatment on 15 June 2023 (**Figure 2B**) at a hospital. During the period of receiving the above therapy, the patient was repeatedly reexamined for neck, chest, and abdomen CT (**Figures 2C, D**), which all indicated that she had stable disease (SD). In order to control the right sacroiliac metastasis, radiation therapy was administered followed the chemotherapy and targeted therapy (5Gy×5f).

Given the first-line treatment response (SD), the adverse reactions of bone marrow suppression of chemotherapy (grade 3), and the results of genetic testing, targeted treatment of almonertinib (110 mg per day), combined with glumetinib (150 mg per day), was administered as a second line of treatment on 14 November 2023. 20 days later, the examination for neck, chest, and abdomen CT (**Figure 2E**) indicated SD. Due to the prevalence of novel coronavirus pneumonia, the patient did not receive regular reexamination. However, 77 days later after taking almonertinib and glumetinib (29 January 2024), she developed a fever as high as 39.4°C, accompanied by cough, expectoration, and shortness of breath after exercise. Intermittent hormone and anti-infection treatments were given at a hospital, but there was no significant improvement in the above symptoms. To our satisfaction, the reexamination for neck, chest, and abdomen CT (**Figure 2F**) on 5 February 2024 indicated partial response (PR). Based on the above information, the attending doctor considered that her fever was related to glumetinib and suggested stopping treatment with glumetinib. However, after stopping glumetinib on 20 February 2024, the patient still had an intermittent fever as high as 39.5°C.

**Figure 3:**
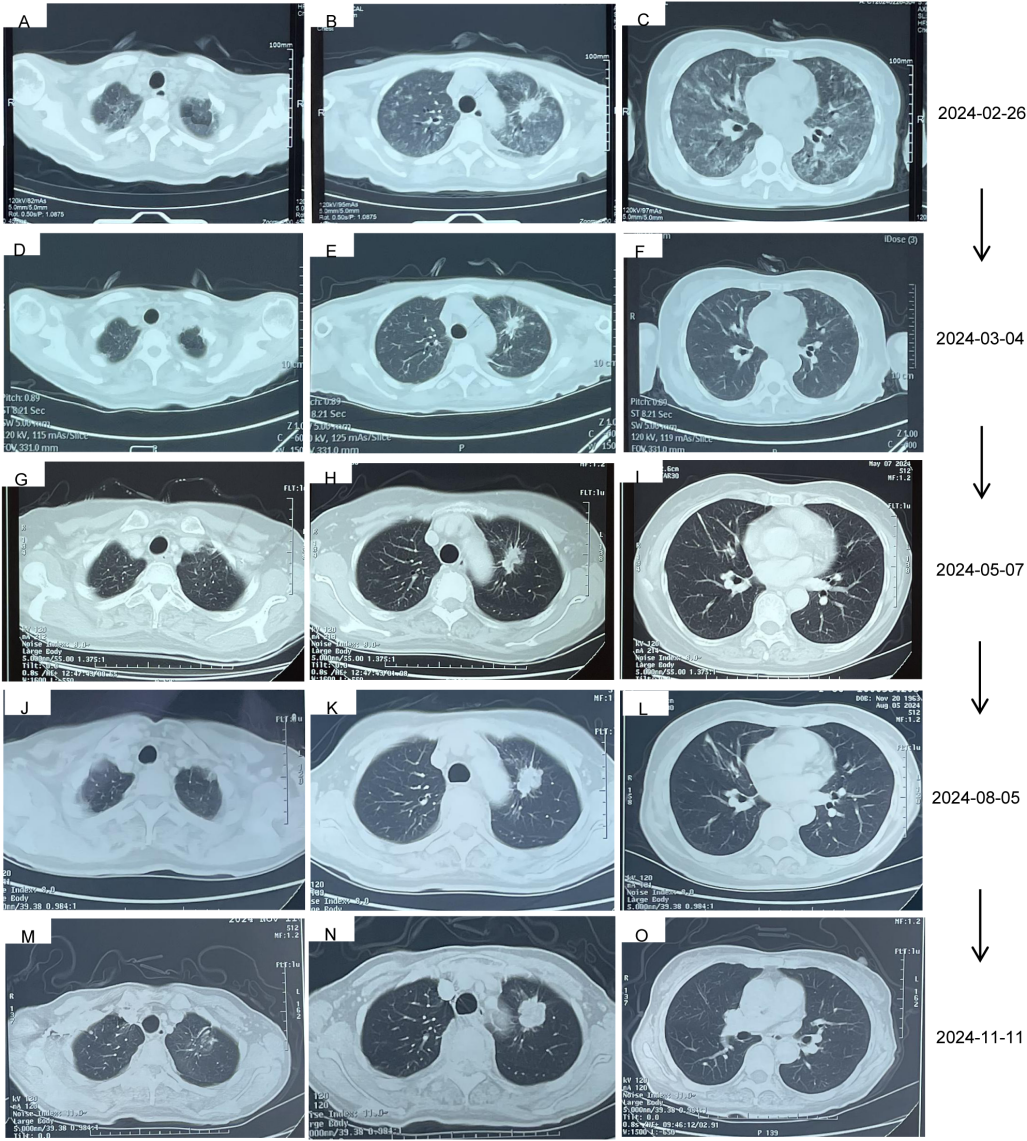
The ILD changes of the patient. **(A–C)** 105 days after the treatment of almonertinib, 99 days after the treatment of glumetinib, and stopped taking glumetinib for 6 days. **(D–F)** The ILD has been well controlled after he anti-infection and methylprednisolone (40 mg/day) for 8 days. **(G–I)** 50 days after the treatment of furmonertinib, 22 days after the treatment of furmonertinib and glumetinib without recurrence of ILD. **(J–L)** The ILD has been well controlled at Aug, 5, 2024. **(M–O)** The ILD has been well controlled at Nov, 11, 2024.

Thus, she was admitted to our hospital for further treatment on 23 February 2024. A series of relevant laboratory examinations were requested (**Table 1**). However, the patient was unexpectedly found to have severe shortness of breath and difficulty in breathing on 26 February 2024 (**Figures 3A–C**). Moreover, inspiratory crackles were heard over the lower zones of the lung. The emergency chest CT indicated diffuse ILD along with lung infection (**Figure 2G**). We organized related discussions, and almonertinib-induced ILD was considered in the absence of other potential causes, so she stopped taking almonertinib by our proposal. After methylprednisolone (40 mg/day, 8 days) along with oxygen uptake, she was supplemented with calcium tablet and gastric mucosal protection. The respiratory condition gradually improved, and chest CT also showed a noticeable improvement in the interstitial lung disease and lung infection on 4 March 2024 (**Figures 2H**, **3D–F**), so the methylprednisolone was decreased gradually, and completely discontinued on 6 May 2024. Based on the ILD which has been well controlled, the attending doctor suggested to choose furmonertinib (80 mg per day, targeted to the EGFR Ex19del mutation) on 19 March 2024, and follow-up for 1 month showed that the patient did not feel any discomfort. Thus, glumetinib was rechallenged on 16 April 2024. To our satisfaction again, although this patient has not received sufficient antitumor treatment for a considerable period of time (57 days), the reexamination for neck, chest, and abdomen CT (**Figures 2I**, **3G–I**) on 7 May 2024 still indicated SD.

**Table 1:** Demographic characteristics and laboratory and imaging findings of the patient.

Demographic characteristics	
Age-yr	60
Gender	Female
Smoking history	No
Initial findings on admission to our hospital	
Past medical history	Almonertinib, glumetinib
Primary symptoms	fever, cough, expectoration, shortness of breath after exercise.
Laboratory findings on admission to our hospital	
White blood cell count (10 ^9^ /liter)	4.05
Neutrophils (%)	54.1
Eosinophils (%)	1.8
Lymphocytes (%)	27.8
Hemoglobin (g/liter)	92
Platelet count (10 ^9^ /liter)	107
C-reactive protein (mg/liter)	47.5
Erythrocyte sedimentation rate (mm/hour)	62
Procalcitonin	0.34
Alanine aminotransferase (U/liter)	103.3
Aspartate aminotransferase (U/liter)	63.7
Total protein	44.1
Albumin (g/liter)	22
Creatine kinase (U/liter)	263.4
Myoglobin (ug/liter)	125.1
Creatinine (umol/liter)	55.2
Uric acid (umol/liter)	206.6
Activated partial-thromboplastin (sec)	0.88
Thrombin time	16.9
Fibrinogen (g/liter)	3.23
D-dimer (mg/liter)	0.31
(1,3)- β- D-glucan concentration (pg/ml)	26.6
Antinuclear antibody series	Antinuclear antibody, Cytoplasmic antibody, centromere antibody, anti-dsDNA antibody, anti-nRNP antibody, anti-Sm antibody, anti-SS-A antibody, anti-SS-B antibody, anti-Scl-70 antibody, anti-Jo-1 antibody, anti-rRNA antibody, anti-mitochondrial-M2 antibody, anti- histone antibody, anti-centromere antibody, anti-proliferating cell nuclear antigen antibody,anti-nucleosome antibody, anti-Ro52 antibody, anti-PM-Scl antibody, anti-dsDNA antibody were negative.
Culture of bacteria (urine, sputum, stool)	Negtive
Culture of fungi (urine, sputum, stool)	Negtive
2019 Novel Coronavirus nucleic acid	Negtive
Multiple virus testing	Human respiratory syncytial virus antibody, Adenovirus antibody, influenza virus A antibody, influenza virus B antibody, Parainfluenza virus 1-4 antibody were negative.
C. pneumoniae antibody	Negtive
M. pneumoniae antibody	Negtive
L. pneumophila1-12 antibody	Negtive
Multiple Respiratory pathogen (nucleic acid)	Streptococcus pneumoniae, Staphylococcus aureus, methicillin-resistant Staphylococcus aureus, Klebsiella pneumoniae, Pseudomonas aeruginosa, Acinetobacter baumannii, Stenotrophomonas maltophilus, Haemophilus influenzae, Pneumocystis pneumoniae, Mycoplasma pneumoniae, Chlamydia pneumoniae, Mycobacterium tuberculosis complex, Legionella pneumophila, Cartamola pneumoniae, Nocardia, Chlamydia trachomatis, Chlamydia psittaci were negative.
Pathogen identification using bronchoalveolar lavage fluid (metagenomics next-generation sequencing)	Coving more than 25000 Pathogenic microorganisms: Bacteria (11836), viruses (11021), fungi (1872), parasites (421), rickettsia (105), mycoplasma/chlamydia (118), mycobacteria (153), and only found Human Herpesvirus 4 positive.
Image findings on admission to our hospital	
chest CT on Feb, 26, 2024	diffuse interstitial lung disease (ILD) along with lung infection.
chest CT on Mar, 4, 2024	improvement in the interstitial lung disease and lung infection compared to Feb, 26, 2024.

The reexamination for neck, chest, and abdomen CT (**Figures 2J**, **3J–L**) on 5 August 2024 indicated SD. The rebiopsy of the sacroiliac bone (right side) performed on 30 August 2024 indicated TTF-1(3+), PDL1(22C3) (TPS:3%), and PDL1(22C3Neg) (−), and the molecular screening of sacroiliac bone biopsy tumor tissue by a large gene new-generation sequencing (NGS) panel analysis identified EGFR 19 exon p.L747-P753 mutation (38.6%), TP53 exon8 p.V272L mutation (40.7%), CD8 exon2 p.A50D (13.5%), WPN exon33 p.A1297G (2.3%), IL7R exon3 p.N106I mutation (14.6%), and CCNE1 (CN=3.9) amplification. Then, targeted treatment of sunvozertinib (150 mg per day) combined with glumetinib (150 mg per day) was administered as a third line of treatment om 14 September 2024. The reexamination for neck, chest, and abdomen CT (**Figures 2K**, **3M–O**) on 12 November 2024 indicated SD but showed a slow progress trend, so it was adjusted to sunvozertinib (150 mg per day) combined with vinorelbine (30 mg biw).

At the time of this writing (2 January 2025), the patient was still without recurrence of ILD (**Table 1**).

## 3 Discussion

We reported a case of initially diagnosed advanced lung adenocarcinoma co-harboring EGFR Ex19del mutation and MET *de novo* amplifications (**Table 2**). The effectiveness of the front-line pemetrexed and carboplatin chemotherapy along with bevacizumab (six cycles) was limited. However, the patient responded positively to the second-line dual-targeted therapy of almonertinib (110 mg per day), combined with glumetinib (150 mg per day), but ILD was found 3 months later. After anti-infection and methylprednisolone (40 mg/day, 8 days) along with oxygen uptake, calcium tablet and gastric mucosal protection was supplemented. The respiratory condition gradually improved, and chest CT also showed a noticeable improvement. Up to now (2 January 2025), the patient is still without recurrence of ILD.

**Table 2:** The treatment for the patient.

The case timeline	The treatment for the patient
From mid-Jun, 2023 to mid-Nov, 2023	Six cycles of front-line pemetrexed and carboplatin chemotherapy along with bevacizumab-targeted therapy, bisphosphonate bone protection treatment and radiation therapy.
From Nov, 14, 2023 to late-Jan, 2024	Targeted treatment of almonertinib (110mg per day, targeted to the EGFR Ex19del mutation) combined with glumetinib (150mg per day, targeted to the MET de-novo amplifications) as a second line of treatment.
In late-Feb, 2024	Stopping treatment with glumetinib due to intermittent fever as high as 39.5°C fever and received methylprednisolone (40 mg/day, 8 days) along with oxygen uptake, supplemented by calcium tablet and gastric mucosal protection.
From Mar, 4, 2024 to May, 6, 2024	Methylprednisolone was gradually reduced and completely discontinued.
From mid-Apr, 2024 to mid-Sep, 2024	Gemetinib treatment again after 1 month observation of taking furmonertinib (80mg per day, targeted to the EGFR Ex19del mutation) without any discomfort.
From mid-Sep, 2024 to mid-Nov, 2024	Sunvozertinib (150mg per day) combined with glumetinib (150mg per day) was administered as a third line of treatment.
From mid-Nov, 2024 to present	Sunvozertinib (150mg per day) combined with vinorelbine (30mg biw)

Although EGFR-sensitive mutation is very common in the Asian population, and MET amplification as a *de novo* driver alteration occurring in untreated NSCLC patients is approximately 1%–5% (7, 8), the co-harboring EGFR Ex19del mutation and MET *de novo* amplification is really rare (18, 19). Thus, a unified standard treatment plan has not been formed. Although the first-line dual-targeted regimens are not routinely recommended for this group of patients, the treatment of dual-targeted (second-line) was more effective than carboplatin chemotherapy along with bevacizumab (first-line) for the patient we reported. Furthermore, the patient we reported felt that the overall tolerance process of dual-targeted therapy was better than carboplatin chemotherapy along with bevacizumab. Unfortunately, the dual-targeted treatment was stopped due to ILD.

Antineoplastic agent-induced ILD, which was primarily associated with chemotherapy, targeted therapy, and immunotherapy, is as the primary cause (23%–51%) of drug-induced ILD (20). Although risk factors vary among different antineoplastic agents, physicians also should carefully evaluate the risk for ILD before the start of any anticancer therapy, which is available at https://doi.org/10.1016/j.esmoop (20). Usually, men, who smoke, who are 55 years old and above, and with pneumopathy (chronic obstructive pulmonary disease, history of interstitial pneumonia, pulmonary infectious diseases), presence of contralateral pulmonary metastasis, normal lung area less than 50%, and combined heart disease, were more likely to confront with ILD (21–24). However, the risk of drug-induced ILD increases when causative drugs are used in combination, and for some drugs, can be dose-dependent (20). The risk factors for the patient we reported were the combination of targeted drugs (almonertinib and glumetinib) and advanced age. At present, the mechanisms of EGFR-TKI-induced ILD are not yet completely understood. The reported mechanisms including preventing the regeneration and proliferation of damaged epithelium, inhibiting protein kinase B and extracellular signal-regulated kinase (ERK) 1/2, and activating p38 mitogen-activated protein kinase (MAPK) disrupt the balance of cell survival, producing the cytokine interleukin-6 (IL-6) and so on (22, 25). Usually, the above risk factors are unavoidable, but the progression of ILD can be well controlled in time through early judgment and intervention.

As antineoplastic agent-induced ILD can be difficult to identify and manage, and in most cases only sporadically (26), the relevant experience of most doctors is insufficient, and currently there are no specific guidelines on the diagnosis and treatment of it. It is recommended that physicians should use the Pneumotox online platform (https://doi.org/10.1016/j.esmoop.2022.100404) to know the risk of ILD before antineoplastic agent therapy. Once ILD is suspected, multidisciplinary interaction is very important in the diagnosis and management of targeted drug-induced ILD. The symptoms of ILD are generally non-specific, with the most frequent being non-productive cough, asthenia, and chest pain. Dyspnea, low-grade fever, cough, fatigue, and chest pain and tightness should be carefully evaluated, and dyspnea on exertion is the most important symptom to be alert to with the occurrence of ILD (27). Physical examination and careful patient history-taking (in order to obtain detailed information on the drugs taken by the patient, comorbidities, and any potential risk factors, as well as to rule out any other cause of ILD and to define the temporal relationship between the onset of symptoms and exposure to the potentially causative drug) (28), measurement of vital signs (especially respiratory rate, arterial oxygen saturation, and abnormal pulmonary auscultation may detect alterations in the normal vesicular murmur and typical pulmonary crackles), relevant laboratory tests, respiratory function tests (a baseline assessment with these tests should be carried out as soon as drug-induced ILD is suspected, which shows a restrictive spirometric pattern with a decline in total lung capacity and should be repeated over time to monitor respiratory function), and lung diffusion capacity for carbon monoxide and computed tomography are the important components of an accurate diagnosis, at the same time, although microbial and serological testing are not specific, but could help to exclude or confirm infectious causes (viruses, bacteria, fungi, and so on). High-resolution CT (HRCT) is currently the most sensitive diagnostic modality for detecting ILD since its early stages, and a follow-up CT scan should be repeated along with assessment of therapeutic response (26); the CT features are areas of ground-glass opacity (GGO), consolidation and lung volume reduction (29–31), and the corresponding pathological features which are thickening of the alveolar walls, deposition of hyaline membranes, and infiltration of inflammatory cells. Up to one-third of patients with drug-induced ILD can be asymptomatic, so incidental diagnosis in patients with radiological evidence of interstitial pneumonia may occur (32). For the patient we reported, the HRCT taken on 5 February 2024 has shown signs of ILD, and the clinical symptoms at that time were fever, cough, expectoration and shortness of breath after exercise. The attending doctor considered that her fever was glumetinib-induced ILD, so stopping of treatment with glumetinib was suggested. However, the progression of ILD was not alleviated by discontinuing glumetinib. When she came to our hospital, the patient did not tell us that her chest CT suggested ILD, and she did not know she had ILD; her main complaint was a fever of unknown cause, so a series of relevant laboratory examinations have been taken (**Table 1**), excluding lung inflammation, virus infection, rheumatism, tuberculosis, cardiac failure, and so on. Moreover, until the patient was unexpectedly found to have severe shortness of breath and difficulty in breathing on 26 February 2024 and the emergency chest CT indicated significant progression of ILD, a diagnosis of almonertinib-induced ILD was determined, so almonertinib was urgently stopped. Then, the family sent the chest CT results taken on 26 February 2024, and we found that ILD had already existed at that time. The judgment and intervention of ILD in this patient was not timely, which emphasized careful patient history-taking and vigilance for targeted-induced ILD contributed to the judgment of targeted-induced ILD timely. Otherwise, it will cause serious consequences.

Almonertinib, a new third-generation EGFR-TKI, was approved by the National Medical Products Administration as first-line treatment of locally advanced or metastatic NSCLC with 19Del and 21L858R mutation on 16 December 2021. Moreover, the main advantage is almonertinib and its metabolites have weak inhibition on wild-type EGFR, so there are fewer side effects (33). A higher proportion of adverse events with almonertinib are rash and elevation of creatine phosphokinase, aspartate aminotransferase, and alanine aminotransferase; ILD was extremely rare. ILD was only observed in the cohort receiving 260 mg in the phase I study (34), no ILD was reported in the phase II study (APOLLO) (35), and only two cases of ILD were observed in the phase III study (AENEAS) (36). Up to now, there are only two case reports of almonertinib-induced ILD: one was reported in 2020 by Ting Jiang (a 70-year-old woman, 110 mg per day, 3 months later ILD was found) (37); another was reported in 2023 by Xiaokui Tang (a 71-year-old man, 110 mg per day, 3 months later ILD was found) (38). In addition, Longqiu Wu reported a case of osimertinib-induced ILD and then switched to almonertinib for further treatment with success (39). Probably based on the above report and experience, the attending doctor considered the ILD of the patient we reported to be glumetinib induced, so pausing of treatment with glumetinib was first suggested, but almonertinib was continued. Furthermore, for the patient we reported, 77 days after taking almonertinib and glumetinib, she developed a fever as high as 39.4°C, accompanied by cough, expectoration, and shortness of breath after exercise. The time of occurrence almonertinib-induced ILD was shorter than previously reported, which may be due to the aggravation of toxic and side effects of dual-targeted therapy (almonertinib combined with glumetinib). However, from the perspective of the whole diagnosis and treatment process, we are more inclined to almonertinib-induced ILD. Although the risk of drug-induced ILD increases when causative drugs are used in combination (26), the presence of glumetinib-induced ILD cannot be completely ruled out.

As recommended in the review of “Drug-induced interstitial lung disease during cancer therapies: expert opinion on diagnosis and treatment” (26), the treatment approach in case of drug-induced ILD mainly consists in the discontinuation of the offending drug and start of immunosuppressive therapy and is always driven by the grade of severity of the clinical manifestations. In grade 3 ILD, hypoxic patients should receive oxygen therapy according to the degree of hypoxemia, and the timely and definitive discontinuation of the anticancer drug and the initiation of corticosteroid therapy at 1 mg/kg/day–2 mg/kg/day of methylprednisolone or equivalent are essential. If the patients respond well and revert to grade 1 (complete resolution of the symptoms with possible persistence of the radiological features), steroid therapy can be progressively tapered after 8–12 weeks; however, rapid steroid de-scalation may increase the risk of ILD reactivation (26). For the patient, we reported methylprednisolone (40 mg/day) along with oxygen uptake for 3 days; the patient felt her respiratory condition gradually improved, and chest CT also showed a noticeable improvement in the ILD on 4 March 2024, so the methylprednisolone was decreased gradually and completely discontinued on 6 May 2024.

After the remission of almonertinib-induced ILD, it is necessary to choose appropriate drugs to control tumor progression. Although we did not obtain the rechallenge recommendations of EGFR-TKI drug after almonertinib-induced ILD, most literatures confirm that when an EGFR-TKI is discontinued due to ILD, replacing other EGFR-TKI drugs can usually successfully control tumor progression (40–42). For the patient we reported, based on the ILD which has been well controlled, the attending doctor suggested to choose furmonertinib, which, as a novel, third-generation EGFR-TKI, is safe and well tolerated in NSCLC patients with EGFR-sensitive mutations and EGFR T790M-resistant mutations to control tumor progression. Moreover, follow-up for 1 month showed that the patient did not feel any discomfort. Thus, glumetinib was rechallenged. According to the changes of her condition, medication was adjusted to sunvozertinib (150 mg per day) combined with glumetinib (150 mg per day) on 14 September 2024. Then, it was adjusted to sunvozertinib (150 mg per day) combined with vinorelbine (30 mg biw) on 11 November 2024. However, there were no adverse drug reactions such as ILD, and the patient did not feel any discomfort either.

As a whole, for this patient, the effectiveness of dual-targeted therapy was the highest. Besides ILD, the patient did not feel any other discomfort. As targeted therapy may induce new mutations or cause the disappearance of existing targets, re-biopsy was thus necessary and the occurrence of ILD should always be looked out for.

## 4 Conclusion

This study reports that patients with EGFR Ex19del mutation and MET *de novo* amplification may benefit more from dual-targeted therapy than pemetrexed and carboplatin chemotherapy along with bevacizumab. However, dual-targeted therapy may increase the risk of ILD, so it is important to be alert to targeted-induced ILD, and unexplained fever may be an early warning signal for targeted-induced ILD, especially almonertinib-induced ILD. Timely intervention is needed to avoid greater harm when ILD occurs and, when ILD is effectively controlled, seize the opportunity to rechallenge the dual-targeted therapy, which may contribute to a better prognosis. In addition, the patients with targeted-induced ILD in the past need more rigorous monitoring and follow-up in the process of rechallenging the targeted drug therapy.

## Data Availability

The original contributions presented in the study are included in the article/supplementary material. Further inquiries can be directed to the corresponding authors.
